# A Pasteur Case

**Published:** 1889-08-24

**Authors:** 


### Within the Wards.

### A PASTEUR CASE.

Notwithstanding the admitted weight of the great names, both British and foreign, which may be counted on the side of M. Pasteur and his hydrophobia treatment, a rigid scientific induction declares that the treatment is in no sense a fully demonstrated success, but is still *sub judice*. The following case, which was published in the *Illustrated Medical News* of November 17, 1888, is particularly interesting to all students of the subject at the present moment. Mr. Victor Horsley, an ardent Pasteurian, read a paper at the meeting of the Clinical Society of London on Friday, November 9, 1888. The paper owned the joint authorship of Dr. Bristowe, F.R.S., senior physician to St. Thomas's Hospital, and Mr. Horsley himself, and recorded the principal facts of a case of "paralytic rabies in a man bitten by a mad cat." The man was treated locally at the time of the bite, and subsequently sent to M. Pasteur. On his return from Paris he developed obscure symptoms resembling those of perityphlitis, or inflammation in the region of the large bowel. The following day he complained of pain in the back, and the day afterwards was seized with acute ascending paralysis, from which he speedily died. There seems to have been no doubt in the minds of the medical men that the cause of his death was rabies, and this was considered to be conclusively proved by an inoculation which was made from his spinal cord. Mr. Horsley described further researches and experiments, from which it appeared that the virus of the rabid cat was especially powerful. Not only so, but the period during which this particular virus is, or appears to be, latent in the system is very short—as short, it is said, as that of the pure virus isolated by M. Pasteur. But if that be really the case, it would seem to be quite futile to treat the bites of mad cats by the Pasteur method even an hour or two after the bite. Dr. Bristowe expressed the conviction that the disease, notwithstanding its curious course and manifestations, was due to the bite, and not to the inoculation, though he admitted that that point was quite open to discussion. Mr. Horsley was asked why the inoculations in this case had completely failed to protect the man. His defence was exceedingly instructive. It consisted practically of an array of statistics. It is true, he urged, that paralytic rabies would not follow inoculation if the animal had been inoculated in the first instance with non-virulent matter; though how that argument bears upon the question of the failure of the treatment to protect or in any other way influence the patient for good no ordinary mind can perceive. He did further add the opinion—which, of course, was only an opinion, and must be taken for what it is worth—that some persons, like some animals—rabbits, for instance—were so susceptible to the disease that they could not be rendered proof against it, especially if they were infected by the virus of the cat. He claimed that Pasteur's statistics of mortality (2 per cent.) contrasted very favourably with the results of bites of dogs recognised (by whom?) to be mad elsewhere—in Lancashire, for example, where 15 per cent. of the cases terminated fatally. The indisputably protective effect of prophylactic inoculation was, he said, inferentially in favour of the treatment. This may be taken as a fair sample of the strongest arguments which Pasteurians can bring forward when they are face to face with men as scientifically trained as themselves. The reasoning proves beyond a doubt that Pasteur's method requires to be much more extensively tested, by a great many critical minds, under a variety of circumstances, before it can be accepted as a distinct gain to practical medicine. A statistical argument, though valuable when the statistics are gathered from many and various sources, is too tainted with suspicion to be of great weight when all the statistics on one side of the question come from one source only, and that a deeply interested source. We admire Pasteur and much of his work greatly, as it deserves to be admired; but we submit that true science has no respect for names and authorities, but only for definite, demonstrable, and undeniable facts.

